# Courtship behavior of the castniid palm borer, *Paysandisia archon* : potential roles of male scents and visual cues in a day-flying moth

**DOI:** 10.1093/jis/14.1.52

**Published:** 2014-01-01

**Authors:** Roxane Delle-Vedove, Brigitte Frérot, Martine Hossaert-McKey, Laurence Beaudoin-Ollivier

**Affiliations:** 1 Centre d’Ecologie Fonctionnelle et Evolutive (CEFE), UMR CNRS 5175, 1919 route de Mende, 34293 Montpellier cedex 5, France; 2 CIRAD, UPR Bioagresseurs, Avenue Agropolis F-34398 Montpellier, France; 3 INRA Versailles, UMR PISC 1272, Route de St Cyr 78026 Versailles Cedex, France

**Keywords:** behavioral sequences, Castniidae, mating, male pheromones, mate choice

## Abstract

The castniid palm borer, *Paysandisia archon* (Burmeister) (Lepidoptera: Castniidae), is a South American moth that in the last ten years has become a major pest of palm trees in the Mediterranean region. Current knowledge on the reproductive behavior of this diurnal moth suggests the importance of both visual and chemical cues, in particular the production of a male pheromone emitted during a specific scratching behavior. Male-produced scents have diverse functions in lepidopteran sexual communication but generally act during courtship behavior, leading to complex, stereotyped courtship sequences. As a first step to understand the cues involved in mating behavior and the role of male scents in male mating success, we quantified sequences of *P. archon* courtship behavior using video filming. To distinguish behaviors leading to an approach of both partners from those involved in short-range courtship, sequences were divided into “approach” and “interaction” phases. Quantifications and analyses were first made by NPMANOVA analysis of behavioral event frequencies, followed by flowchart construction using transition matrix probabilities. In 90% of the observations, courting activities led to copulation, but successful sequences were highly variable and could be divided into two categories, “rapid” and “prolonged” courtship sequences. In both categories, approaches were performed by males but depended strongly on female movements, especially on female flights. The significant behavioral differences were observed after the first contact (i.e., interaction phase) where, in rapid sequences, males generally acceded to copulation without displaying scratching behavior. Conversely, in prolonged sequences, the female expressed evading behavior and male scratching frequency increased. The possible roles of male scent emission in female mate choice and the importance of visual cues in the mating behavior of *P. archon* are discussed.

## Introduction

Sexual communication in Lepidoptera involves use of visual, acoustic, and olfactory signals. In nocturnal moths, chemical signaling, especially the production of specific female pheromones, is central for mate detection and localization. Female long-range attractants have received much attention in the past decades, as they are at the core of efficient methods for pest population monitoring ( Cardé and Minks 1995 ). Conversely, in day-flying butterflies, visual signals are primarily used for both the detection of females by males and the assessment of mating partners by females ( Jiggins et al. 2001 ; Papke et al. 2007 ). These different signals are not exclusive and may be displayed at the different stages of mate recognition and choice rather than constituting a complex behavioral sequence leading up to mating ( Baker and Cardé 1979 ; Klein and Araujo 2010 ). In particular, males of several (diurnal) butterfly species are known to produce short-range pheromones, which have recently received much attention in efforts to understand their role in mate selection by females ( Costanzo and Monteiro 2007 ; Papke et al. 2007 ; Nieberding et al. 2008 , 2012). For example, in the butterfly *Bicyclus anynana* , male-produced chemicals have been found to act in combination with visual cues to influence mate choice by females ( Costanzo and Monteiro 2007 ). To date, however, no evidence exists that day-flying moths use both visual and chemical cues to assess mate quality, but males of some day-flying zygaenid moths are known to use both visual and chemical cues to detect females ( Toshova et al. 2007 ).

Males of some moth species also have elaborate scent organs (androconia, hairpencils, brushes, etc.) associated with specific behaviors allowing diffusion of a male courtship pheromone. The biological roles of moth male pheromones are diverse and sometimes still controversial (see reviews by Birch et al. 1990 ; Svensson 1996 ). Male emissions may act as an aphrodisiac, arresting female departure and enhancing their acceptance of copulation ( Birch et al. 1989 ; Andersson et al. 2007 ). It has also been suggested that male pheromones are involved in female mate choice by giving indirect information about male quality ( Phelan and Baker 1986 ; Iyengar et al. 2001 ). In moth species where males possess specific scent organs, courtship behavior has been found to be particularly complex and ritualized, allowing males to bring scent organs in close proximity to the female antennae ( Phelan and Baker 1990 ). For example, in *Grapholita molesta* , after the male lands near the female, it extrudes abdominal hairpencils and propels the pheromone over the female’s head, attracting her at short distance ( Baker and Cardé 1979 ; Baker et al. 1981 ). It has been suggested that such ritualized courtship behaviors may have evolved to increase the efficiency of pheromonal message delivery and may, in addition to pheromone composition, ensure the reproductive isolation of species ( Phelan and Baker 1987 ). In this context, the description of courtship sequences represents the first necessary step to understand not only the cues that may be involved in mate recognition and choice, but also the potential role of male scents in male mating success.

The family Castniidae is a small moth family found in the neotropics and Australia, with a few species in southeast Asia. The family belongs to the Sesioidea and includes large diurnal moths familiarly called “butterfly moths” ( Kristensen et al. 2007 ; Rios and Gonzales 2011 ). The reproductive behavior of castniids remains largely unknown. However, efforts to understand its behavior have been recently spurred by the accidental introduction of the castniid palm borer, *Paysandisia archon* (Burmeister) (Lepidoptera: Castniidae), originating from South America, into the Mediterranean area, where it became invasive. Larvae of this species are internal feeders on palms, and may cause severe damage, particularly at the sites of introduction, where it is considered a pest. Adults of *P. archon* do not feed; both sexes display colorful underwings with no evident sexual dimorphism ( Sarto I Monteys and Aguilar 2005 ). It has recently been suggested that male *P. archon* may exhibit butterfly-like mating behavior with visual detection of females by males and production of a short-range pheromone by males ( Sarto I Monteys et al. 2012 ). According to observations by these authors, two major phases of the *P. archon* mating behavior may be defined: (1) the visual detection of the approaching flying female by a perching male, and (2) the male courtship behavior itself, ending with copulation. Nevertheless, these authors provide no detailed quantifications of the behaviors, especially for behaviors linked to male pheromone emission that could give insight into the role of these chemicals in the species’ mating behavior. Moreover, the conclusions of Sarto I Monteys et al. (2012) have recently been contradicted by the identification of a female pheromone in the ovipositor of sexually mature *P. archon* females and of a male pheromone not present in wing extracts as previously purported by Sarto I Monteys et al. (2012) , but associated with androconia on the midlegs ( Delle-Vedove 2011 ; Frérot et al. 2013 ). Both male and female pheromones were found to be associated with a specific scent emission behavior: the typical “calling” in females ( Ollivier and Frérot 2006 ) and the so-called “scratching” behavior of males, during which the insect scratches its midlegs on the substrate, suggesting the release of chemicals contained by androconia ( Frérot et al. 2013 ).

In this little-studied context of a brightly colored day-flying moth in which both sexes release pheromones, our study set out to quantify courtship behavior and examine in particular the potential roles of (1) of visual cues in the mating behavior and (2) male androconial pheromones by analyzing the occurrence of scratching behavior in the sequence. To answer these questions, we conducted outdoor experiments under abiotic conditions close to those experienced by this moth in nature. Based on the framework of *P. archon* courtship behavior proposed by Sarto I Monteys et al. (2012) , we focused experiments on the two main phases of courtship to describe behaviors involved in (1) detection and approach and (2) short-range interactions leading to copulation. To understand the part each of these phases played in determining male mating success, we also compared quantitatively the differences between successful and unsuccessful courtships.

## Materials and Methods

### Insects

The insects used in our experiments were collected as last-instar larvae or pupae from infested palms that were felled in the Montpellier area (France, 43.37°N, 3.52°E) between January and July in 2010 and 2011. After collection, larvae were placed in individual circular plastic boxes (80 mm diameter x 50 mm height) with organic matter collected from damaged palms and kept in the dark in a warehouse under natural temperature conditions (ranging from 5°C to 30°C) until pupation. Larvae pupated between April and August, and pupae were kept in the laboratory at 25°C under a natural photoperiod. Each day, the sex of emerging moths was determined. Males and females were kept in fine mesh cages (30.5 x 30.5 x 30.5 cm, www.livemonarch.com ) under the same conditions as indicated above and in separate rooms to avoid exposing them to odors of the other sex. To ensure that insects used in the experiments were sexually mature, they ranged in age from at least one day old (maximal time required for newly emerge adults to reach sexual maturity; Delle-Vedove et al. 2012 ) up to six days.

### Behavioral observations

Observations were carried out under outdoor conditions on sunny days from June to August in 2010 and 2011, always between 11:00 and 16:00 (the period of peak sexual activity, Delle-Vedove et al. 2012 ). As the emission of the female pheromone is generally necessary to initiate male mating behavior in moths ( Charlton and Cardé 1990 ; Girling and Cardé 2006 ), one virgin female was placed in a fine mesh cage (61 x 61 x 91 cm ^3^ ) provided with a young host palm ( *Chamaerops humilis* L. (Arecales: Arecaceae), 40-60 cm high), and observed until the onset of calling behavior. Then, a randomly chosen virgin naive male was released into the cage. A camera (Canon Legria HF-200, www.canon.com ) was used to record behavior from the introduction of the male to the end of copulation or to 16:00 (end of the sexual activity period, Delle-Vedove et al. 2012 ) when copulation did not occur. A total of 40 pairs of imagos were observed, leading to 29 hr of courtship observations. Cages were cleaned with a solution of De-con90 (1%) ( www.decon.co.uk ) after each experiment to avoid contamination by chemicals produced by male and/or female moths. Six additional variables that might influence mating were also recorded: age of the individuals, duration of the pre-calling interval before introduction of the male, and abiotic conditions (temperature, light, and wind speed).

### Video analyses and behavioral ethogram

The videos of the 40 courtship sequences were watched in real time or frame-by-frame when necessary using PMB software (Sony, www.sony.com ). A total of 20 behavioral events were defined to describe the behavior of males and females ( Table 1 ). To discriminate between behaviors leading to an approach and those involved in short-range interaction, each courtship sequence was divided into two different phases, approach phase and interaction phase. All the sequences began by an approach phase leading to a contact between the two individuals. The interaction phase started with this first contact, followed by an interaction in which individuals were separated by less than 10 cm. When this arbitrary defined distance was exceeded for more than one minute, individuals were considered to enter a new approaching phase.

**Table 1.**
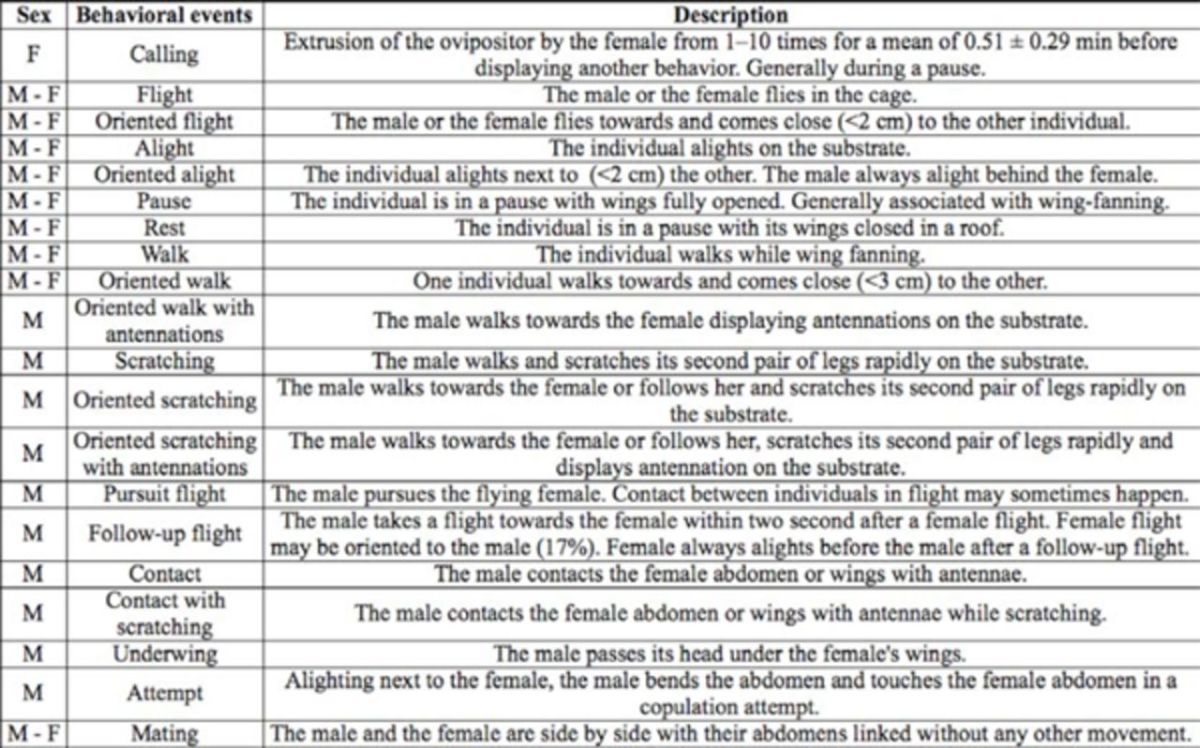
Description of behavioral events involved in the courtship behavior of *Paysandisia archon.*

### Behavioral quantification and statistics

Behavioral quantifications and statistical analyses were performed to test for behavioral differences between successful and unsuccessful courtship sequences. Nevertheless, great variation in the number of interaction phases (and so in the number of approach phases) was observed before mating, suggesting that it was more appropriate to define two other courtship categories, “rapid” and “prolonged” (see Results for a description). Quantifications and statistical analyses were then performed to test for behavioral differences between these two categories using two different methods.

First, the absolute frequencies of each behavioral event were calculated independently for the first approach and interaction phase of each courtship sequence. To test for significant differences in frequencies of events between rapid and prolonged courtships, two non-parametric analyses of variance (NPMANOVA) were performed independently for each type of phase. Absolute frequencies of each previously defined behavioral event were ranked and taken as a response variable in the NPMANOVA (Finch 2005; Mega and Araujo 2010 ). When the NPMANOVA proved significant, the univariate Mann-Whitney test was performed as a *post-hoc* test for differences in the frequency of specific behaviors. To avoid statistical deviations due to small sample sizes, behavioral events with low frequencies (<1 %) were excluded.

Second, to elicit the most probable behavioral transitions and build behavioral flowcharts of the courtship behavior, each sequence was converted to a transition probability matrix (Guirling and Cardé 2006; Mega and Ajauro 2010 ). These matrices were calculated for each male, with the preceding behavioral event in rows and the succeeding event in columns. To avoid underestimations of transition probabilities, self-transitions of a single behavioral event were not recorded. Transition frequencies for each male were then averaged across all males of a courtship category (rapid or prolonged) to produce a unique matrix for each category. The most probable transitions were then used to build behavioral flowcharts using Microsoft Powerpoint 2007 ( www.microsoft.com ).

To test whether differences between the two courtship categories could be explained by differences in experimental conditions, and as abiotic variables were found to be not normally distributed, an NPMANOVA was performed on the six additional variables mentioned above (age of the individuals, duration of female calling before introduction of the male, temperature, light, and wind speed). All the analyses were done using R 2.12.0 (R Development Core Team).

## Results

### Description of behavioral events

In all, 20 behavioral events were defined, of which nine were shared by both sexes ( Table 1 ). Most of these events amounted to classical displacement or resting behaviors (pause, walk, flight, and alight). To understand which behaviors were involved in the approach of an individual to that of the other sex, in each case it was noted whether the event was oriented or not towards the other individual ( Table 1 ).

One behavioral event was displayed by females only: calling, during which the female extruded her ovipositor ( Table 1 ). Calling was observed in both the approach and interaction phases of the courtship sequences, but also when females were alone in cages before introduction of a male.

Ten behavioral events were observed in males only ( Table 1 ). Four of these involved scratching behavior, which was observed in both approach and interaction phases of the courtship sequences. The follow-up flight was also typically displayed by males and characterized by the male making an oriented flight (towards the female) immediately after a female flight ( Table 1 ). Apart from these behaviors, most of the male-specific behaviors were only displayed during the interaction phase and represented typical courtship (i.e., sexual) behaviors.

### Categorization of courtship sequences

Of a total of 40 sequences, 90% resulted in successful mating, but great variation in the number of interaction phases before mating was found. This led to a separation of the courtship sequences into two categories, named rapid and prolonged, described below.

Rapid courtship sequences (62.5%; n = 25): In these sequences, mating success was 100%, and mating was rapidly achieved. The mean duration between introduction of the male and mating was 15.30 ± 7.75 min. Only one interaction phase (and one approach) was observed before mating.

Prolonged courtship sequences (37.5%; n = 15): In these sequences, the number of observed interaction phases before mating was significantly higher than for rapid courtship sequences (5.09 ± 2.98, Mann-Whitney: *U* = 4, *P* < 0.01). Consequently, the time from introduction of the male to mating was also greater (54.66 ± 30.76 min), significantly higher than for rapid courtship sequences (Mann-Whitney: *U* = 282, *P* < 0.01). The mating success in this category was 73% (no mating occurred within the observation period for four of 15 pairs).

The occurrence of rapid or prolonged courtship was not related to experimental conditions, as no significant variation in the additional variables (age of individuals, female calling duration, or abiotic conditions) was found between the two courtship categories (NPMANOVA: F-test = 1.075, *P* = 0.366).

**Figure 1.**
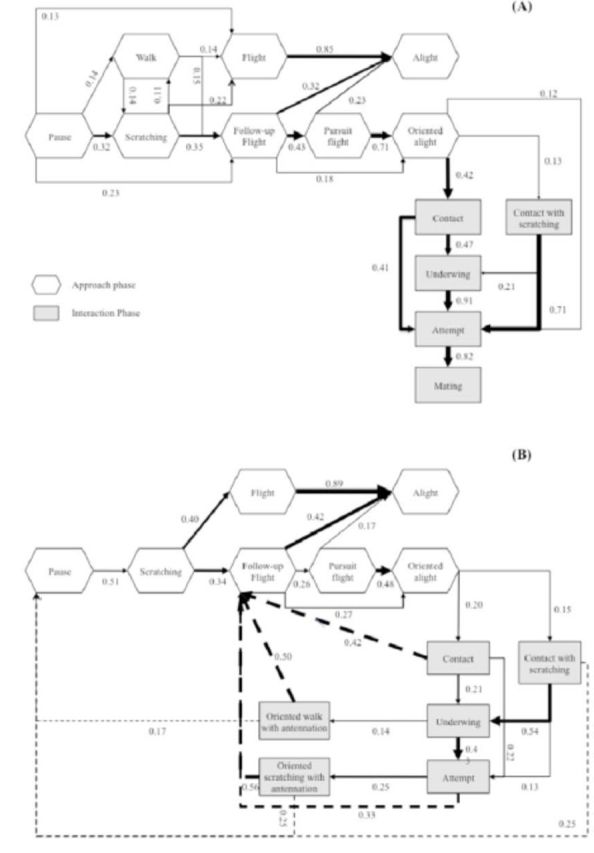
Behavioral flowcharts of the male behavior in the first approach (white boxes) and interaction (gray boxes) phases of *Paysandisia archon* courtship. Flowcharts are constructed from mean transition probabilities for (A) “rapid” courtship sequences (n = 25) and (B) “prolonged” courtship sequences (n = 15). Decimal numbers and the corresponding thickness of arrows represent probabilities of a particular transition between two behavioral events. Dotted arrows indicate behavioral transitions that return back to earlier behaviors of the sequence. To enhance clarity, only returns from the interaction phase to the approach phase are represented, and transitions with a value less than 0.1 are not included. Descriptions of behavioral events are listed in Table 1 . High quality figures are available online.

**Behavioral sequence of mating behavior Approach phase.** Both sexes were found to display scent-related behaviors (calling and scratching) during this phase ( Table 2 ). The comparison of absolute frequencies of the behaviors exhibited during approach did not reveal significant differences for either male or female behavior between prolonged and rapid courtship sequences (NPMANOVA: F-test = 0.961, *P* = 0.422). Females were found to be highly active in the cage and alternated walk, flight, and pause with calling ( Table 2 ), but these displacements were not oriented to the male and thus did not lead to encounters between the individuals. Analysis of behavioral transitions revealed that the male follow-up flight, followed by an oriented alight, was the only significant sequence leading to contact and interaction between males and females for both rapid and prolonged courtship sequences ( Figure 1 ). The follow-up flight, which was highly dependent on preceding female flight ( Table 1 ), represented the typical male approach behavior.

**Table 2.**
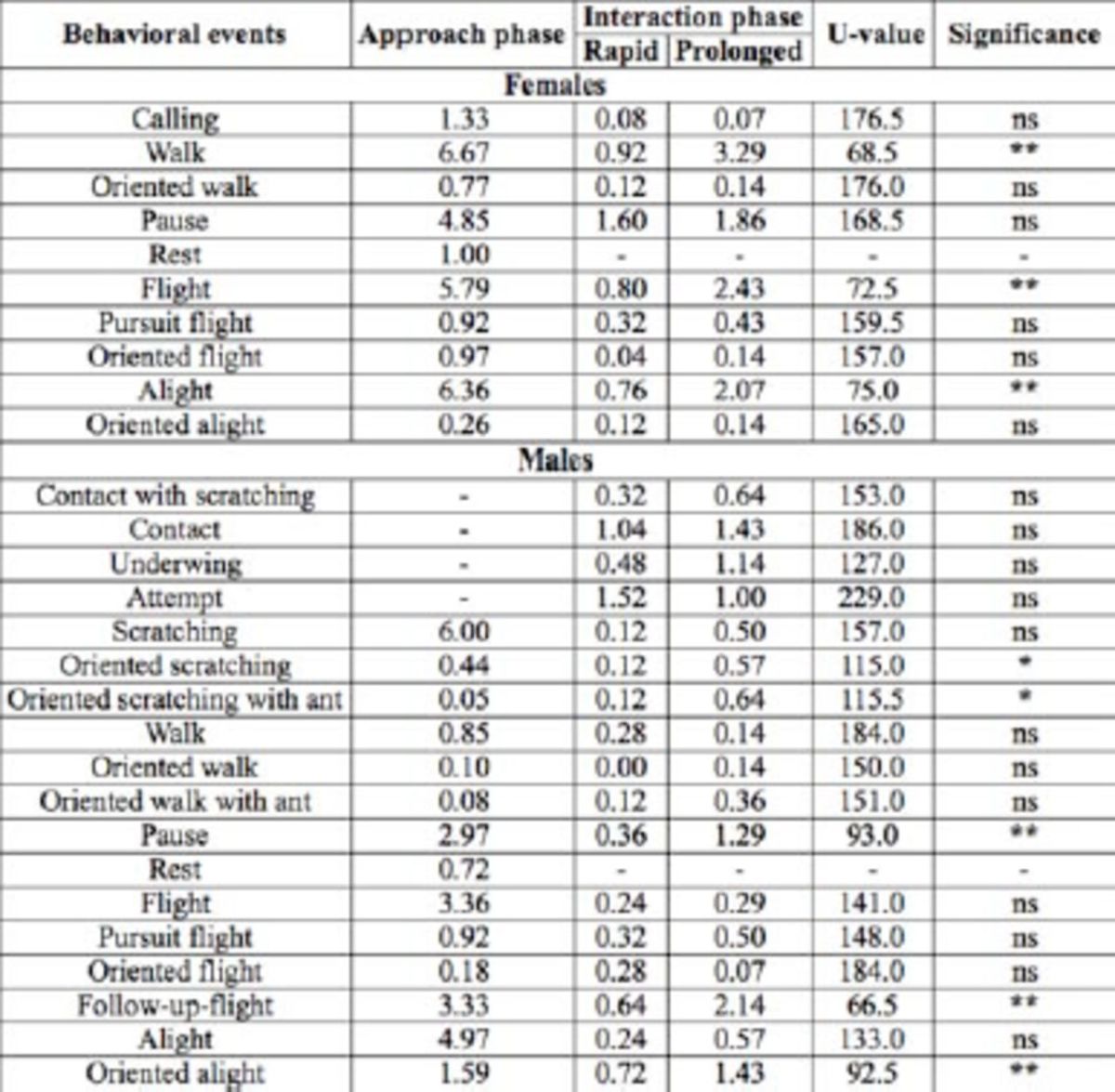
Mean absolute frequencies of behavioral events displayed by *Paysandisia archon* during the first approach and interaction phases of courtship sequences. As no significant differences between “prolonged” and “rapid” courtship categories were found during the approach phase (see Results), data for the approach phase are pooled across the two categories. Results of the *post-hoc* analysis (U value and significance) performed to test for behavioral differences between “rapid” and “prolonged” courtship in the interaction phase are indicated.

U value and significance refer to the univariate Mann-Whitney test for each behavioral event of the interaction phase. ns = not significant; ^*^ , *p* < 0.05; ^**^ , *p* < 0.01

**Interaction phase.** After the male approached, the pair alighted, led by the female, and short-range courting activities were observed. Significant behavioral variation between rapid and prolonged courtships was found during this phase (NPMANOVA: F-test = 10.26, *P* < 0.01). In rapid courtship sequences, females were mostly inactive, as a pause was the most frequently observed behavior ( Table 2 ), allowing males to perform their courtship. Contrary to our expectations on the role of male scent in courtship, scratching behaviors were generally not observed during this phase in rapid courtship sequences and mating success most often followed the behavioral succession of contact, underwing, and attempt behaviors ( Figure 1A ). However, mating success was achieved more rapidly when males performed an attempt directly after landing next to the female ( Figure 1A ).

In prolonged courtships, we observed an overall significant increase in female activity (walk, flight, and alight; Table 2 ). Nevertheless, no increase in behaviors oriented to the male (oriented walk or oriented flight) was observed, indicating a female tendency to move away from males. This female “male-evading” behavior was observed frequently after the first contact with the male, as exem-plified by the high frequency of returns to the approach phase after the first contact behavior ( Figure 1B ). As a result, the contact between individuals was interrupted several times during the interaction phase. Nevertheless, the distance between individuals was minimized, because a significant increase of the typical male approach behavior (follow-up flight and oriented alight) was also observed ( Table 2 ). In addition, we observed a significant increase in male scratching behaviors (oriented scratching and oriented scratching with anten-nations; Table 2 , Figure 1B ). These male and female behaviors constitute a so-called “pursuit loop” represented in the behavioral flowchart by a high level of returns from the interaction to the approach phase (dotted arrows; Figure 1B ).

## Discussion

The results highlight a stereotyped male approach, dependent on female flight (follow-up flight), leading to a large proportion (90%) of observed copulations. Contrary to expectations on the role of male scent in mediating short-range courtship, male scratching was generally observed before the male approached the female. These results raise the question of the role of visual cues and of male scent in the detection of potential mates in *P. archon* . Despite the fact that this sequence generally led to an interaction between the partners, great behavioral variation in the interaction phase (i.e., short-range courtship behavior) was found. In most of the sequences, males succeeded rapidly in copulating (rapid courtship sequences) without scent emission during short-range interaction. In other cases, however, males were observed to pursue the evading female and to display scratching behavior prior to copulation (prolonged courtship sequences). We will discuss the potential role of male-produced chemicals in female choice.

### General behavioral sequence leading to an approach

In most moth species, males actively seek mates in a patrolling behavior, while females display few behaviors, generally restricted to calling before the male approach, and acceptance or rejection of the male during short-range interactions ( Charlton and Carde 1990 ; Girling and Carde 2006 ). Males detect the stationary calling females, alight near them, and start courting activities ( Charlton and Carde 1990 ; Girling and Carde 2006 ). In contrast, in *P. archon* , females were not stationary before a male approach, and female flights were necessary for their detection by a male, initiating approach by the male in a follow-up flight. Following this behavior, a pursuit flight by both individuals was frequently observed, and the pair alighted on the substrate, led by the female. Courtship in *P. archon* thus involves flight by both partners. This sequence is in accordance with observations by Sarto I Monteys et al. (2012) of males perching on palms and taking flight towards and chasing flying objects that pass in front of them. These results strongly suggest that *P. archon* relies on a perching mate-finding strategy rather than a patrolling one.

Female flights may be perceived by males simply as a movement, but may also provide them with more specific visual signals. Indeed, *P. archon* adults have colorful hindwings, visible during the major part of the courtship sequences; rest is the sole behavioral event during which they are hidden, and this behavior was observed infrequently. In diurnal butterflies, visual cues are involved in mate localization and recognition ( Wiklund 2003 ; Costanzo and Monteiro 2007 ), but this is also known in some day-flying moths. For example, in brightly-colored zygaenid moths, visual cues, in combination with chemical cues, have been found to be important in mate location by males ( Naumann et al. 1999 ; Toshova et al. 2007 ).

Contrary to expectations about the role of male scent as a courtship pheromone, male scratching was observed before the approach, but also when males were alone in cages (R. Delle-Vedove, unpublished data). These observations suggest that the role of male scent is not restricted to courtship and may be involved in searching and locating conspecific males. In some moth species, male scent has been found to act as female attractant to male aggregation ( Birch et al. 1990 ), or in a dual mate-finding strategy, as in *Trichoplusia ni,* in which females are attracted to male scents both in wind-tunnel experiments and in the field ( Landolt and Heath 1990 ; Landolt 1995 ). In this species, two different mate-finding strategies are used, depending on the period of scotophase, one relying on the attraction of females to males, and the second, more common, in which females call to attract males ( Landolt and Heath 1990 ). As both sexes display scent emission behavior, our study suggests a dual mate-finding strategy may characterize *P. archon* as well.

### Male courtship success

Once a male had approached a female, there was great variation in subsequent courtship behavior, in particular the occurrence of a so-called pursuit loop in prolonged sequences. This loop was characterized by the female displaying male-evading behaviors, while the male, in turn, responded with more oriented behaviors, particularly the typical approach behavior, follow-up flight. Similar variation has been found in *Amyelois transitella,* where prolonged successful courtships also involve, more interruptions in interactions before copulation, with females displaying evading behavior ( Girling and Cardé 2006 ). In *P. archon* , the occurrence of interruptions in interactions led to a 73% chance of copulation, but that proportion would probably be lower under natural conditions, where females have a greater chance of avoiding males than in a closed experimental environment. The observed female refusal may reflect reduced receptivity of the female. In our experiment, all the females were sexually mature, but receptivity is often affected by the quality and persistence of males ( Rutowski 1982 ; Wedell 2005 ). We thus surmise that this female behavior reflects female mate selection, expected to be particularly stringent in a monandrous species, such as *P. archon,* and that likely involves responses to visual, chemical, acoustic, and/or tactile signals ( Svensson 1996 ).

In rapid successful sequences, most of the behaviors, especially contact behaviors, are often skipped from the sequence. Some males successfully copulated after an attempt behavior only. Thus, mate selection by females may be independent of contacts with males and linked to signals emitted by males and perceived by females before the male approaches. Male pheromones may be important in female mate choice in some Lepidoptera ( Phelan and Baker 1986 ; Iyengar et al. 2001 ; Nieberding et al. 2012 ). For example, in *Utetheisa ornatrix* , females mate preferentially with certain males on the basis of their emission of hydroxydanaidal, a courtship pheromone ( Iyengar et al. 2001 ). Pheromone emission behavior by male *P. archon* was always displayed before the male approached the female and not during the short-range interactions in rapid successful sequences. There was no significant variation in the male scent release effort, as the frequencies of male scratching in the approach phase between “rapid” and “prolonged” sequence were not different. We might expect males to differ quantitatively and/or qualitatively in their chemical message and, as in *U. ornatrix* , females may choose among males on that basis. Nevertheless, in two of the rapid courtship sequences observed, some individuals displayed no scratching behavior during the entire sequence but nevertheless succeeded in mating. This suggests that other signals, visual or perhaps acoustic, may be associated with chemical signals in determining the female’s choice of mate, as in the day-flying butterfly *Bicyclus anynana* , where visual and chemical cues have been found to be of equal importance in female mate choice ( Costanzo and Monteiro 2007 ).

Males that were assumed here to be “less attractive” to females generally showed specific behaviors during interactions. In particular, they pursued the females by displaying more frequent scratching behavior. Indeed, male pheromones may also act during interactions as a signal to increase female receptivity in the case of initial refusal. Increased frequency of scratching by the male might lead to increased quantity of pheromone emitted, which might be a factor in female choice. The evading behaviors of females may also be a form of mate quality assessment in which the male is required to perform continued orientation and maintain contact with the female, its success in doing so affecting female mate choice ( Wedell 2005 ; Girling and Cardé 2006 ).

### Evolution of mating behavior in Castniidae

Courtship behavior, along with pheromone composition, is part of the specific recognition system that enables reproductive isolation of species occurring in sympatry. Contrary to expectations about courtship behavior in moth species in which males have scent-producing organs ( Baker and Cardé 1979 ; Phelan and Baker 1990 ), the short-range courtship behavior of *P. archon* does not involve obligate specific scent-emission behaviors. Indeed, the shortest way to achieve copulation, in rapid courtship, was much simpler, with males only displaying an attempt not preceded or accompanied by scent-emitting behaviors. Castniid moths are mainly brightly-colored, day-flying moths and may mimic and occur sympatrically with representatives of butterflies. Visual cues, which seem from our results to be of great importance in the mating behavior of *P. archon* , may reflect the evolution of specific recognition cues more adapted to its diurnal lifestyle period. In butterflies, females have no long-range pheromones. Sarto I Monteys et al. (2012) did not detect any female pheromone or scent-producing glands and concluded that *P. archon* is characterized by a butterfly-like mate-finding strategy. Nevertheless, the extraction of female ovipositors at the time of sexual maturity has recently permitted the identification of a female pheromone in *P. archon* ( Delle-Vedove 2011 ), and our results have clearly shown that female *P. archon* exhibit calling behavior. Determining whether calling behavior is associated with long-range attraction requires further studies using wind-tunnel experiments. However, our results, in contrast with those of Sarto I Monteys et al. (2012) , suggest this species is characterized by a “moth-butterfly” hybrid strategy relying on both chemical and visual cues.

### Conclusions

The results reported here provide the first quantified knowledge of the close-range mating behavior in castniids, and highlight a specific strategy in which both chemical and visual cues are displayed. In the experiments, courtship usually led to successful copulation, but some males succeeded rapidly whereas females tended to avoid others. For the former, copulation did not involve a complex and stereotyped courtship, as might have been expected from studies of courtship behavior in other moth species whose males produce scent ( Birch et al. 1989 ; Baker et al. 1981 ; Phelan and Baker 1990 ). Males generally did not display scratching behavior during short-range interaction, except when faced with an evading female. We presume from these results that male scents may act as quantitative signals affecting female mate choice, acting primarily before the male’s approach and secondarily during subsequent interaction to enhance receptivity of females that attempt to evade males. The data also highlight that, unlike the usual case in moths, female movements, especially flights, were necessary to initiate male approaches. This result may reveal the importance of visual cues in the mating behavior of *P. archon* and is suggested to be linked to a perching male mate-finding strategy, typical of day-flying butterflies and already observed in *P. archon* ( Sarto I Monteys et al. 2012 ). The use of classical lure-and-kill applications for the control of this pest may be limited by the apparently important role of visual cues in mate location. We nevertheless suggest that both male and female sex pheromones might be involved in a dual mate-finding strategy, such as that already observed in another moth ( Landolt and Heath 1990 ). The possible application of pheromones in disruption of mating needs further investigation. By decoupling chemical and visual cues, wind-tunnel experiments could enhance understanding of the mate-finding strategies of this diurnal moth and notably of the role of the female calling behavior and of male pheromones as primary attractants. Further studies should also investigate variation in male pheromone content and its role in female mate choice by studying the copulation success of males without scent structures and/or supplemented with a controlled dose of male pheromone.
